# Mental health, physical impairment and violence among FSWS in North Karnataka, South India: a story of intersecting vulnerabilities

**DOI:** 10.1192/bjo.2021.636

**Published:** 2021-06-18

**Authors:** Alicja Beksinska, Tara S Beattie, Lucy Platt, Parinita Bhattacharjee, Ravi Prakash, Satyanarayana Ramanaik, Kavitha Dibbadahalli, Martine Collumbien, Mitzy Gafos, Calum Davey, Charlotte Watts, Shajy Isac, Rachel Jewkes

**Affiliations:** 1 Department of Global Health and Development, London School of Hygiene and Tropical Medicine; 2 Department of Public Health, Environments and Society, London School of Hygiene and Tropical Medicine; 3 Karnataka Health Promotion Trust; 4 Karnataka Health Promotion Trust, University of Manitoba, Department of Community Health Sciences; 5 Gender and Health Division, South African Medical Research Council

## Abstract

**Aims:**

This study examines the prevalence and associations between recent violence experience, mental health and physical health impairment among Female Sex Workers (FSWs) in north Karnataka, India.

**Background:**

Multi-morbidity, in particular the overlap between physical and mental health problems, is an important global health challenge to address. FSWs experience high levels of gender-based violence, which increases the risk of poor mental health, however there is limited information on the prevalence of physical health impairments and how this interacts with mental health and violence.

**Method:**

We conducted secondary analysis of cross-sectional quantitative survey data collected in 2016 as part of a cluster-RCT with FSWs called Samvedana Plus. Bivariate and multivariate analyses were used to examine associations between physical impairment, recent (past 6 months) physical or sexual violence from any perpetrator, and mental health problems measured by PHQ-2 (depression), GAD-2 (anxiety), any common mental health problem (depression or anxiety), self-harm ever and suicidal ideation ever.

**Result:**

511 FSWs participated. One fifth had symptoms of depression (21.5%) or anxiety (22.1%), one third (34.1%) reported symptoms of either, 4.5% had ever self-harmed and 5.5% reported suicidal ideation ever. Over half (58.1%) reported recent violence. A quarter (27.6%) reported one or more chronic physical impairments. Mental health problems such as depression were higher among those who reported recent violence (29%) compared to those who reported no recent violence (11%). There was a step-wise increase in the proportion of women with mental health problems as the number of physical impairments increased (e.g. depression 18.1% no impairment; 30.2% one impairment; 31.4% ≥ two impairments). In adjusted analyses, mental health problems were significantly more likely among women who reported recent violence (e.g. depression and violence AOR 2.42 (1.24–4.72) with rates highest among women reporting recent violence and one or more physical impairments (AOR 5.23 (2.49–10.97).

**Conclusion:**

Our study suggests multi-morbidity of mental and physical health problems is a concern amongst FSWs and is associated with recent violence experience. Programmes working with FSWs need to be mindful of these intersecting vulnerabilities, inclusive of women with physical health impairments and include treatment for mental health problems as part of core-programming.

Samvedana Plus was funded by UKaid through Department for International Development as part of STRIVE (structural drivers of HIV) led by London School of Hygiene and Tropical Medicine and the What Works to Prevent Violence Against Women and Girls Global Programme led by South African Medical Research Council

